# Age, glucose tolerance, and cognitive performance in vervet monkeys

**DOI:** 10.1002/alz70855_106968

**Published:** 2025-12-24

**Authors:** Brett M. Frye, Trinity G Davis, Thomas C. Register, Suzanne Craft, Mark G. Baxter, Carol A. Shively

**Affiliations:** ^1^ Wake Forest Alzheimer's Disease Research Center, Winston‐Salem, NC, USA; ^2^ Wake Forest School of Medicine, Winston‐Salem, NC, USA; ^3^ Emory and Henry University, Emory, VA, USA; ^4^ Wake Forest University School of Medicine, Winston‐Salem, NC, USA

## Abstract

**Background:**

Alzheimer's disease (AD) is the primary cause of dementia worldwide and is increasing in incidence. In humans, impaired glucose handling characteristic of pre‐ and type 2 diabetes (T2D) is associated with cognitive dysfunction and AD risk. Vervet monkeys (*Cholorocebus aethiops sabaeus*) have emerged as a nonhuman primate model of early AD due to similarities to humans in phylogeny, endocrine, sociality, cognition, brain structure and function, AD‐like neuropathology, and response to risk factor modification. Thus, these NHPs provide important opportunities to determine relationships between age, glucose handling, and cognitive performance.

**Method:**

We determined glucose and insulin levels in 101 ivGTTs, conducted over 4 yrs (∼16 human years), in 41 middle‐ to very old‐aged female vervets (9‐30 years), living in long term, stable social groups. We determined temporal relationships between glucose handling and age, and cognitive performance (executive function and working memory), and examined BMI as a modifier of these relationships.

**Result:**

Cluster analyses yielded four distinct patterns of glucose handling and insulin responsivity relevant to the development of T2D: 1) normal insulin response and euglycemia, 2) hyperinsulinemia with baseline euglycemia, 3) lack of insulin response and euglycemia, and 4) lack of insulin response and hyperglycemic. Age was associated with baseline glucose (*r*(51)=0.57, *p* <0.001), insulin area under the curve (AUC) from 0‐60 minutes (*r*(35)=‐0.27, *p* = 0.017), insulin AUC from time 10‐40 minutes (*r*(31)=‐0.23, *p* = 0.031), glucose AUC from time 10‐40 minutes (*r*(48)=0.43, *p* = 0.030), and glucose clearance from time 10‐40 minutes (*r*(42)=‐0.48, *p* <0.001). Older age plus higher BMI predicted poorer glucose handling (r(69)=0.96, *p* = 0.055). Glucose handling was associated with subsequent cognitive performance. Executive function declined with decreasing insulin production (F(1,9)=7.61, *p* = 0.022) in middle‐age, whereas working memory declined with increasing glucose levels at older ages (glucose baseline: F(1,10)=21.13, *p* <0.001; AUC F(1,10)=12.72, *p* = 0.005). Older vervets with better glycemic control had better cognitive function than their same aged counterparts with poorer glycemic control.

**Conclusion:**

Vervets exhibit biological subtypes of glucose and insulin physiology like humans, and develop age‐related impaired metabolic functioning. Glucose handling predicts cognitive performance; these relationships may manifest in older age and vary with obesity. Funding: R24AG073199, Harry O. Parker Neuroscience Research Fund, P30AG072947

